# Supplementary material for: Comparative Analysis for Genetic Characterization in Korean Native Jeju Horse
Source: Animals (Basel). 2021 Jun 28;11(7):1924. doi: 10.3390/ani11071924 (PMC8300358; doi:10.3390/ani11071924)
Supplement: Supplementary file 1 [file animals-11-01924-s001.zip › Supplementary_Figure_S1.pdf]

Supplementary Figure S1.

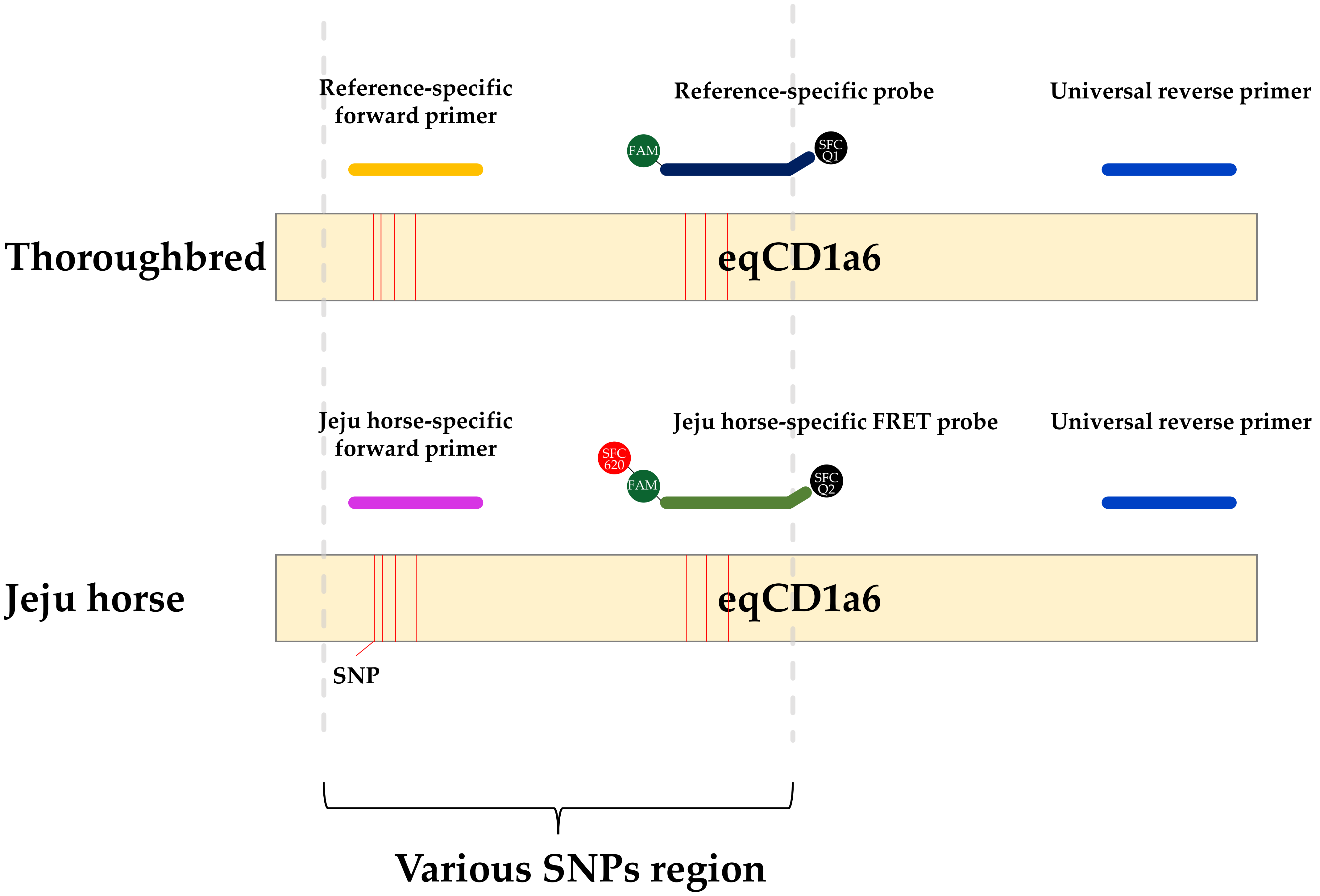

RSF: CAGTGAGGAGCTGACTGAAATCG
JSF: CAGCAATAAGGAGATGACTGAAATGG
UR: CAAGCTGCCATTGACTGGCAT
JSP: FAM/ATCCCGTATGACCTCCATTGGATTTAATCAGG/SFCQ1
RSP: SFC620/AGT/FAM/ATTCCGAACGTTCAGCATTGAATTTAATCAGA/SFCQ2

RSF = Reference-specific forward primer
JSF = Jeju horse-specific forward primer
UR = Universal reverse primer
JSP = Jeju horse-specific probe
RSP = Reference-specific FRET probe
